# Book Reviews

**Published:** 1852-01

**Authors:** 


### BIBLIOGRAPHICAL NOTICES.

*Lehrbuch der physiologischen Chemie.* Von Prof. Dr. C. G. Lehmann. Zweite gänzlich neu umgearbeitete Auflage. 8vo. Erster Band, S. 464. Zweiter Band, S. 463. Leipzig, 1850.

*Manual of Physiological Chemistry.* By. Prof. Dr. C. G. Lehmann. Leipz., 1850.

*Handbuch der physiologischen und pathologischen Chemie nach den neuesten Quellen:* bearbeitet von Dr. A. Moser und Dr. J. C. Strahl. 8vo. S. 759. Leipzig, 1851.

*Manual of Physiological and Pathological Chemistry, after the most recent sources.* Prepared by Dr. A. Moser and Dr. J. C. Strahl. Leipzig, 1851.

*Physiologie des Stoffwechsels in Pflanzen und Thieren.* Ein Handbuch für Naturforscher, Landwirthe und Aerzte, von Dr. Jac. Moleschott, Privatdocentem der Physiologie an der Universität zu Heidelberg. 8vo. S. 592. Erlangen, 1851.

*Physiology of the Interchange of Matter in Plants and Animals.* A Manual for Naturalists, Agriculturists, and Physicians. By Dr. Jac. Moleschott, &c., &c. Erlangen, 1851.

*Familiar Letters on Chemistry in its relations to Physiology, Dietetics, Agriculture, Commerce, and Political Economy.* By Justus Von Liebig. Third edition, revised and much enlarged. 12mo. pp. 530. London, 1851.

The appearance of the books, of which the above are the titles, is ample evidence of the direction which the investigations of observers are taking in Germany; yet although a greater number of works on physiological and pathological chemistry are issued from the teeming press of that country, it would be scarcely justifiable to infer, that more importance is attached to the subject there than elsewhere. The fact, indeed, that the "Familiar Letters on Chemistry," now before us, have reached a third edition in England, is sufficient to show the direction of the professional and general mind in that country, and if we were possessed of an accurate knowledge of the number of copies of the different works of that distinguished chemist, which have been circulated in this country, we should be astonished at it. To every physician, naturalist, agriculturist, and educated gentleman, the name of Liebig is as familiar as that of Sir Humphry Davy was formerly; and perhaps, amongst the medical profession, the reputation of the former has been even more diffused, owing to the various speculations to which his observations have given rise—on biological subjects more especially.

It may perhaps be esteemed a work of supererogation, at the present day, to expatiate on the advantages of physiological chemistry in elucidating the laws of life, both in the healthy and morbid condition, and yet we are satisfied that, with many, they are overlooked or too lightly regarded, and hence these points are dwelt upon in almost all the treatises of the day on both subjects.

"It is not unworthy of remark," says Liebig, "that many physicians profess to hold chemistry in contempt, exactly as they do with physiology; that medicine reproaches physiology, and with equal injustice as she reproaches chemistry. The physician, who has learned medicine not as a science, but as an em pirical art, acknowledges no principles, but only *rules* derived from experience. The object of his inquiries is only whether a remedy, in any given case, had a good or a bad effect. This is all the empiric cares about. He never asks *why?* He never inquires into the *causes* of what he observes!

"From what a different point of view should we contemplate the abnormal or diseased conditions of the human body; if we were first thoroughly acquainted with its normal conditions, if we had established the science of physiology upon a satisfactory basis. How differently would the treatment of diseases be conducted, if we had perfectly clear notions of the processes of digestion, assimilation and excretion. Without just views of force, cause and effect, without a clear insight into the very essence of natural phenomena, without a solid physiological and chemical education, is it to be wondered at, that men, in other respects rational, should defend the most absurd notions; that the doctrines of Hahnemann should prevail in Germany, and find disciples in all countries. Reason alone will not prevent whole nations from falling into the most abject superstitions, whilst even a child, whose mind has been duly developed and instructed, will repudiate the fear of ghosts and hobgoblins. Can men who do not apprehend the nature of scientific investigation in a philosophical spirit, and who cannot interpret the language of phenomena,—can such men be expected to derive the least advantage from the discoveries of chemistry or physiology; and can they be deemed capable of making the most insignificant application to practical purposes of these discoveries? We often see such persons annoyed, that truth should be so simple, and yet, in despite of all their efforts, they cannot succeed in deriving from it any practical advantages. From such persons emanate the most absurd, nay impossible notions, and they have created for themselves, under the name of the *vis vitæ*, a miraculous theory by which they would explain all the phenomena they are unable to understand. With a totally incomprehensible, indefinable something they would arrest inquiry, and explain every thing which is not incomprehensible. But this *vis vitæ* is itself but a subject of investigation, and in order to explore it, to comprehend its essence, to understand its operations and effects, the physician must pursue the same method, which has been followed in natural philosophy and chemistry with such signal success." p. 14.

Assuredly, it is to chemistry that we are to look for a better acquaintance with many of the phenomena, which are presented by organized bodies; and we only veil our ignorance by words, when we refer them to vital or organic action. In the particular interchange of material that takes place in the composition and decomposition of the tissues, chemistry must play an important part; and the title embraced by Moleschott in one of the works now before us, "*Physiology of the interchange of matter in plants and animals*," is pregnant with meaning. It embraces, in other words, the physiology of chemistry in action in all organized bodies.

It will obviously be inconsistent with the limits of our Journal to attempt an analysis of works so full of detail as those before us. That of Lehmann has long been known, and highly appreciated. The first edition was published nearly ten years ago; and is the source from which much valuable material has been obtained by after inquirers. His experiments on the effect of food of different kinds—animal, vegetable and mixed—on the urine, are familiar to every chemist and physiologist, and his reputation has not been confined to his fatherland, but has extended to every country in which chemical and physiological knowledge has been cultivated. Like Moleschott, Lehmann is not merely an observer, but a philosopher. Not only does he interrogate by experiment, but he observes accurately; and from the observation of phenomena he seeks to ascend to their laws. Hence, the Lehrbuch before us is not simply a detail of facts or phenomena, but an examination into the principles of physiological chemistry in all their applications. With Bacon, he considers that "the true philosopher always seeks to explain and illustrate nature by means of facts, of phenomena; that is, by experiments, the devising and discovery of which is his task, and by which he causes the object of his investigation to speak, as it were intelligibly to him. No single isolated phenomenon taken by itself, can furnish us with its own explanation; but it is by carefully observing and arranging all such facts as are in connection with it, that insight into its nature is attained. For we must never forget that every phenomenon has its reason, every effect its *cause*."—Liebig, p. 15.

After a methodological introduction, in which he depicts the aims and objects of physiological chemistry, and the best means of deriving full advantage from it, Prof. Lehmann inquires into what he terms the "organic substrata of the animal organism." These he investigates in the following order,—the butyric acid group—the succinic acid group—the benzoic acid group—the lactic acid group—the solid fatty acids—the oleaginous fatty acids—the resinous acids—the non-oxygenous alkaloids—the oxygenous alkaloids—the conjugated acids—the haloid bases and haloid salts—the lipoids, (cholesterin, serolin, &c.)—the non-nitrogenous neutral bodies—coloring matters—extractive matters—nitrogenous histogenetic matters (protein combinations) and the mineral substances in the animal body. The consideration of these occupies the whole of the first volume. The second embraces the animal juices, saliva, gastric juice, bile, pancreatic juice, succus intestinalis, fæces, blood, chyle, lymph, transudations, milk, sperm, fluids of the ovum, mucus, secretions of the skin, and urine.

On all these points much valuable information is given, the result of the investigations of the author and others. The acid of the gastric juice he considers to be the lactic, and he accounts for the fact, that free chlorohydric acid passes over in distillation, by the decomposition of the chlorides by the lactic acid at a high temperature; but this does not explain the marked smell of chlorohydric acid, which was observed in every specimen of gastric juice obtained from the Canadian with the fistulous opening into the stomach, who was the subject of the experiments of Dr. Beaumont. Prof. Lehmann, and Messrs. Moser and Strahl detail the discordant views of observers on this matter, which sufficiently exhibit what a discrepancy of opinion still exists amongst chemists in regard to the precise acid that gives the acid character to the gastric juice. It is well known that a nitrogenized body, called pepsin, probably plays an important part in digestion, acting in the same manner as the diastase in plants, and disposing the elements of the food to enter into combination with the gastric acids. Of itself it appears to possess no digestive power, for if the acids be neutralized by alkalies its efficacy is destroyed.—"This," says Dr. Moleschott, "has led Schmidt to the ingenious conjecture that the chlorohydric acid, which was seen by some observers—as Dunglison—to pass over on distilling the gastric juice, is originally paired or conjugated with the pepsin. The conjugated bodies are called by Schmidt chloro-pepso-hydric acid—(Chlorpepsinwasserstoffsaeure.) By strong acids and alkalies, the complex acid, which Schmidt compares to the sulpho-lignic, is decomposed, and in entire accordance with the idea of a conjugated acid, the pepsin which has been thrown down by an alkali does not again unite with its fellow; and hence the digestive property is lost. The same effect is produced by an elevated temperature. At 40° the chloro-pepso-hydric acid, undecomposed, thickens; at 70° the solution becomes turbid; and at 100° the pure pepsin separates in flakes; the fluid portion being dilute chlorohydric acid. Here again, consequently, the efficacy of the conjugated acid is annihilated. Fresh chloro-pepso-hydric acid forms salts with albumen, which, according to Schmidt, are the soluble peptones that arise from the digestion of albuminous substances. Their quantity is limited by the amount of chloro-pepso-hydric acid. They are increased by the addition, in proper limits, of free chlorohydric acid, which decomposes the chloro-pepso-hydric albuminate, forms soluble chlorohydric albumen and sets at liberty fresh portions of the conjugated acid. Gradually, the fluid is saturated with the soluble combinations of albumen, the chloro-pepso-hydric acid is decomposed, and the whole affair is accomplished." p. 424.

This view of Schmidt, is considered by Dr. Moleschott to be the most ingenious and the most explanatory of the phenomena of any yet prepared; and hence he the more regrets that Schmidt has not been able to present, or offer the analysis of any chloro-pepso-hydric salt.

The Handbuch of Physiological and Pathological Chemistry of Drs. Moser and Strahl forms a part of the "Encyclopædia of the Medical Sciences," of which Dr. Moser is the Editor. Like all encyclopedic treatises, it is, of course, a condensed view of the existing condition of the subject, not necessarily enriched by the experiments of the author. It is a valuable exposition, however, which may be consulted with marked benefit. It consists of an introduction, which embraces an inquiry into the properties of organic matters, in contradistinction to the inorganic; the general chemical relations of the animal organism; the analysis of organic matters in general; the necessary apparatus for chemical investigations, &c.; after which are considered, *seriatim*, the anorganic and the organic constituents of the organism; the solids and the fluids; with a brief appendix on the chemistry of disease.

The work of Dr. Moleschott is more comprehensive and more physiological in its scope and character than either of those referred to. The author is accustomed to physiological researches, and is withal an excellent observer and accurate reasoner. His work, as the title imports, is not intended alone for the physician, but for the naturalist and the agriculturist. An enumeration of the titles of the different chapters will convey some idea of the vast amount of important matter embraced in it. They are: the sources of the nutrition of plants; the formation of the generally distributed constituents of plants and animals; history of the generally distributed constituents of plants and animals in the living body; and the disintegration of organic matters after death. Under these heads, the nature of the the soil, air and water; digestion, the chyle, the blood, the tissues, secretions, &c., are investigated, with all the lights that had been shed upon them by former and contemporaneous observers, with whose labors the author had made himself familiar. It is eminently a work that indicates the direction in which the biologist of the day should look for an increase to his knowledge of living phenomena.

The title of the work of Liebig guides us to the nature of the subjects discussed in it. It has been long before the public, and the English translation appears for the third time, with numerous modifications and improvements by its distinguished author. It is a delightful volume, not only for the physician, but for every one who is desirous of forming some acquaintance with the deeply interesting topics of which it treats. It is dedicated to Sir James Clark, and is edited, at the request of the author, by his friend Dr. Gregory; and "from his intimate familiarity with chemical science, and more especially with the physiological subjects treated of, the author is confident that the task could not have been entrusted to better hands." Would that Dr. Gregory had not detracted from the force of this compliment, and injured his fair fame by visionary speculations in the domain of physiology, which compelled us, on a recent occasion, to put the stamp of reprobation on one of his productions, and to regret that he should not have confined himself to chemical facts, with which alone he seems to be really familiar!

To the present edition the author has been induced to prefix a series of letters on the origin and development of chemical science. He has also made large additions in the shape of new letters, containing the results of his researches during the last few years in agricultural and physiological chemistry. The object of dietetics has received from him particular attention, and the "Letters" altogether merit increased attention in the shape in which they are now presented. We know not, indeed, any work of the kind, which is more captivating, and presents greater attractions to the professional and general inquirer. From his first letter we make the following interesting extract, with which we are compelled to conclude.

"It is certainly not conformable to a rational philosophy of nature to attempt an explanation of the processes of formation, nutrition, and secretion in the animal body, before we have obtained a correct knowledge of alimentary substances, and the sources whence they originate, and before albumen, caseine, blood, bile, cerebral substances, &c., had been subjected to a searching investigation. Before these substances have been successfully analyzed, they are mere names, the letters composing which, at the utmost, we know, but whose signification is unknown.

How can it be expected that any useful information should be derived from the mere terms, until the properties and relations of the substances themselves are known; until we have studied the metamorphoses they undergo when in contact with other bodies; until, in short, we have forced them to speak in answer to our questions?

The cause of the phenomena of life is a force, which does not act at sensible distances; its activity becomes manifest only when the aliments or the blood come into immediate contact with the organ destined for their reception or alteration. The chemical force manifests itself precisely in the same manner; indeed, there are no causes in nature producing motion or change in bodies—no powers more closely allied to each other, than the chemical and vital forces. We know that wherever different substances are brought into contact with each other, chemical actions take place. To suppose that one of the most energetic powers of nature should take no part in the processes of the animal organism, although here all the conditions under which it commonly manifests its activity are united, would be against every established rule for the proper study of nature. But so far from there being any foundation for the opinion that chemical force is subordinate to the vital power, so as to become inoperative or imperceptible to us, the chemical effects of oxygen, in the process of respiration, (for example) are seen in full activity during every second of life. Moreover, *urea*, *allantoine*, the acid which is found in *ants* and *water-beetles*, namely *formic acid*, *oxalic acid*, the oils of *valerian root*, of the *spiræa ulmaria*, of the *gualtheria procumbens*, are products of the vital process, &c.; but is their production, we must ask, attributable to the vital force?

We are able to produce all these compounds by chemical processes, that is, by the chemical force. The chemist produces the crystalline substance found in the fluid of the allantois of the cow, from the excrements of snakes and birds: he makes urea from charred blood; sugar, formic acid, and oxalic acid from saw-dust; the volatile oil of *spiræa ulmaria*, of *gualtheria procumbens*, from willow bark; the volatile oil of valerian from potatoes. These results are enough to justify us in entertaining the hope that we shall, ere long, succeed in producing *quinine* and *morphine*, and those combinations of elements of which *albumen* and *fibrine*, or muscular fibre, consist, with all their characteristic properties.

Let us, however, carefully distinguish those effects which belong to the chemical, from those which depend peculiarly upon the vital force, and we shall then be in the right channel for obtaining an insight into the latter. Chemical action will never be able to produce an eye, a hair, or a leaf. But we know, with absolute certainty, that the formation of hydro-cyanic acid and of the oil of bitter almonds, in those seeds, of oil of mustard and of sinapine in mustard, and of sugar in germinating seeds; that all these are the results of chemical decompositions. We see that the stomach of the calf, when dead, with the addition of some hydro-chloric acid, acts upon flesh, and upon coagulated albumen, precisely in the sammer as the living stomach acts; that is, these substances become soluble, and are, in fact, digested. All this justifies us in inferring, that by the scientific investigation of nature, we shall arrive at a clear comprehension of the metamorphoses which alimentary substances undergo in the living organism, and of the action of remedies.

Without a profound study of chemistry and natural philosophy, physiology and medicine will obtain no light to guide them in the solution of their most important problems, that is, in the investigation of the laws of life, the vital processes, and the removal of abnormal states of the organism. Without a knowledge of chemical forces the nature and effects of the vital force cannot be fathomed; the scientific physician can expect to derive assistance from chemistry only when he shall have qualified himself to put his questions to the chemist correctly.

Commerce and the arts have already derived immeasurable advantages from the progress of chemistry; mineralogy has become a new science since regard has been had to the composition of minerals and the chemical relations of their constituents. If the composition and chemical nature of rocks and strata be not in like manner investigated (and this has hitherto been much neglected), it will be impossible to effect any considerable progress in geology. Chemistry, moreover, is the foundation of agriculture, and we cannot hope to give a scientific form and basis to this important art without a knowledge of the constituents of the soil, and of the substances which constitute the food of plants.

Without an acquaintance with Chemistry, the statesman must remain a stranger to the true vital interests of the state, to the means of its organic developement and improvement; his attention cannot become sufficiently alive, nor his perception adequately acute, in regard to what is really useful or injurious to his country,—to society. The highest economic or material interests of a country, the increased and more profitable production of food for man and animals, as well as the preservation and restoration of health, are most closely linked with the advancement and diffusion of the natural sciences, especially of Chemistry.

Without a knowledge of natural phenomena, and of the laws by which they are regulated, the human mind is incapable of forming an adequate conception of the greatness and unfathomable wisdom of the Creator; for all the images which the most inexhaustible fancy or the most cultivated intellect can form will appear, when compared with the reality, but as glittering, variegated, unsubstantial bubbles!

The great desideratum of the present age is practically manifested in the establishment of schools, in which the natural sciences occupy the most prominent place in the course of instruction.

From these schools a more vigorous generation will come forth, powerful in understanding, qualified to appreciate and to accomplish all that is truly great, and to bring forth fruits of universal usefulness. Through them the resources, the wealth, and the strength of empires will be incalculably increased; and when, by the increase of knowledge, the weight which presses on human existence has been lightened, the difficulties of obtaining subsistence lessened, and man is no longer overwhelmed by the pressure of earthly cares and troubles, then, and not till then, will he be able to devote his intellect, purified and refined, to the study of higher subjects of investigation, and finally to the highest of all." p. 20.

